# Integration of Three-dimensional Magnetic Resonance Imaging Spectroscopy with the Leksell GammaPlan Radiosurgical Planning Station for the Treatment of Brain Tumors

**DOI:** 10.7759/cureus.5946

**Published:** 2019-10-20

**Authors:** Fahd Derkaoui Hassani, Adyl Melhaoui, Younes Dif, Abdelhanine Oumoussa, Mohammed Jiddane, Yasser Arkha, Abdeslam El khamlichi

**Affiliations:** 1 Neurosurgery, Cheikh Zaid International Hospital, Center for Doctoral Studies in Life and Health Sciences (CEDoc-SVS), Faculty of Medicine and Pharmacy, Mohammed V University of Rabat, Rabat, MAR; 2 Neuro Oncology - Functional Neurosurgery and Radiosurgery Research Team, Faculty of Medicine and Pharmacy, Mohammed V University of Rabat, Rabat, MAR; 3 Neurosurgery, Center for Doctoral Studies in Life and Health Sciences (CEDoc-SVS), Faculty of Medicine and Pharmacy, Mohammed V University of Rabat, Rabat, MAR; 4 Neuroradiology, Faculty of Medicine and Pharmacy, Mohammed V University of Rabat, Rabat, MAR; 5 Neurosurgery, Faculty of Medicine and Pharmacy, Mohammed V University of Rabat, Rabat, MAR

**Keywords:** 3d mri spectroscopy, metabolic cartography, radiosurgery gamma knife, leksell gammaplan, reverse engineering, software

## Abstract

Introduction

MRI multivoxel spectroscopy mapping is helpful in surgical decision-making. Unfortunately, in daily practice, MRI multivoxel spectroscopy mapping is not always compatible with the current version of Leksell GammaPlan (LGP) (Elekta, Stockholm, Sweden). The aim of this study is to develop a tool to allow the use of this modality in radiosurgical treatments using LGP.

Material and methods

Multivoxel spectroscopy digital imaging and communications in medicine (DICOM) images were analyzed to identify tags to be modified to make the images compatible with LGP. We identify four important tags to be modified for compatibility with LGP. Using Python language, a new software was designed to modify the identified tags and allow the automatic conversion of images to meet LGP requirements.

Results

By modifying the tags of DICOM images, we could use spectroscopic cartography images in radiosurgical planning using LGP. We created a software to reproduce these modifications using a simple and rapid interface. This software executes all the protocols established in the methodology.

Conclusion

The new software, “GP Adapting Solution”, can convert any DICOM image and make it compatible with LGP. The integration of multivoxel spectroscopic images was feasible and could be used for radiosurgical planning. This work is the first step in allowing the potential use of new MRI modalities in radiosurgical planning using LGP. The next steps are to evaluate the impact of these modalities in radiosurgical treatments and to develop methods for integrating other imaging modalities.

## Introduction

Stereotactic radiosurgery (SRS) is a mainstay in contemporary management of brain tumors. The most common indications for the use of SRS are brain metastasis and benign tumors. SRS is sometimes used to irradiate gliomas. An important challenge in this technique is correctly defining the target zone of such an infiltrative lesion [1]. Radiosurgery requires high accuracy and safety. In Gamma Knife radiosurgery, the target is essentially what is visible on T1-weighted MRI and fluid-attenuated inversion recovery weighted MRI. However, spectroscopic MRI (S-MRI) can more accurately define the volume of a lesion with metabolic abnormality [1].

Due to a technical incompatibility with the Leksell GammaPlan (LGP) version 10 (Elekta, Stockholm, Sweden), we could not directly use the metabolic cartography (S-MRI) data on the planning station of the Leksell Gamma Knife Perfexion. To our knowledge, no software will allow this function on this platform. Our purpose is to define the problem and bridge the gap between the data on MRI and LGP. We developed a new software to insert metabolic cartography data on the LGP planning station.

## Materials and methods

For this study, we used a Siemens Magnetom Avanto 1.5-Tesla MRI machine (Siemens Healthineers GmbH, Erlangen, Germany) [2] with a post-treatment station allowing for metabolic cartography on the basis of multivoxel spectroscopy. We also used the Leksell Gamma Knife Perfexion radiosurgery device (Elekta AB, Stockholm, Sweden) [3]. We used LGP version 10, the integrated treatment planning software for Leksell Gamma Knife Perfexion [4].

We applied reverse engineering to the software, which is a process of examining how a software works and drawing useful conclusions from the data. Reverse engineering software code can help find bugs or flaws in need of fixing. Using reverse engineering, we recreate the design of a product by examining the product itself. By this process, we try to understand how the software works by examining what a piece of software does, how it does it, and how we might do the same to reach our goals [5].

Confirmation of the problem

On LGP 10.1, we tried to upload spectroscopic metabolic cartography data but were unable to import these data. We confirmed that LGP 10 does not accept metabolic data. Also, we attempted to use ITK-SNAP (General Public License) [6], software used to segment structures in three-dimensional (3D) medical images, to visualize MRI spectroscopy metabolic cartography. These data could not be visualized on this software. The same was attempted with Osirix (General Public License) but without success. We concluded that there may be an issue with the Digital Imaging and Communications in Medicine (DICOM) data transfer protocol.

By studying different tags using dcmCompare [7], we found that the transfer protocol of DICOM files is related to two tags: Service-Object Pair (SOP) Class and Image Data (ID) study. We manually modified these tags using the Java DICOM (JDICOM) software (Tiani Medgraph, GmbH, Austria) [8]. This modification allowed us to upload those DICOM files on ITK-SNAP and Osirix. Using Osirix, we could create a file called DICOMDir, which indicates the pathway to the concerned files. The DICOMDir is included in the file set and contains the information necessary to read the image files with a DICOM viewer as a separate series [9]. Finally, we could upload these data to the inbox of LGP.

The modification of these tags defining the DICOM transfer protocol allows us to upload the metabolic data. The modifications to the tag parameters made the DICOM files readable by LGP without modifying the data.

Comparison of 3D T1-weighted MRI and S-MRI

The next step was to define tag parameters compatible with LGP and establish a protocol that should be applied to all MRI DICOM images for use in radiosurgical planning. This protocol was divided into three parts. First, we had to define tags mandatory for LGP acceptance, then we had to define tags related to the kind of the MRI sequences (e.g., magnetic proton spectroscopy, functional MRI), and finally, we had to define tags related to the medical images found in the DICOM files. 

The tags used by LGP are those defining the transfer protocol of a DICOM file from one station to another. It contains the type of image and its parameters defined during an MRI or a CT scan. Those tags should be adapted to the LGP software in order to insert data on radiosurgical planning.

We used 22 MRI DICOM image files. We compared the multiplanar-reconstructed 3D T1-weighted MRI that is accepted on the planning station with the spectroscopic cartography but not accepted on LGP. Using dcmCompare, we could estimate the tags that would differ on the platforms. We defined the tags related to this incompatibility, examined each one, and modified the tag without modifying the ID. We defined 73 tags related to the acquisition and transfer of data to LGP.

We can sort these tags into four main categories consisting of general images, image planes, image pixels, and overlay planes. The general images category contains 18 tags related to information about the image (e.g., jpegloss, jpeg lossless) and details of the medical examination. The image plane tags define the image coordinates in space, the resolution, and the pixels and voxels of every single image. The image pixel category consists of five tags defining pixels of a volume, bits of one pixel, and bits reserved to memory. The overlay plane category consists of 18 tags and defines data image display, plans angulation, and image centering. 

This process allows us to understand the role of each tag in importing a DICOM image and its significance in radiosurgical planning on LGP. The principal tags are SOP transfer syntax, photometric interpretation, rows and columns, bits stored, and bits allocated.

Tags modification

We analyzed each tag related to the incompatibility of an MRI DICOM with the LGP station. Then, we modified the parameters of those tags according to the National Electrical Manufacturers Association/American College of Radiology Association standards [10-13]. In performing the modification, we achieved LGP-accepted values. 

Matrix Pixel

This tag represents the image dimensions. Every image is defined by a matrix containing rows and columns. If a DICOM image is not a square matrix, LGP will not accept it.

We created a 512 × 512 square matrix black image and inserted an incompatible and non-square image inside this first one by fusing the centers of both images. This resulted in a newly created square matrix DICOM image without adding any data or modifying the original data.

Image Position Patient

This tag represents the DICOM image coordinates in space. Each image should have a dedicated and unique coordinate. The spectroscopic metabolic cartography is produced on an axial plane. The transition between images is made on the vertical Z plane. This tag is mandatory for LGP to create 3D volume and define the 3D planes. The obtained DICOM images from spectroscopic metabolic cartography do not have coordinates in the vertical plane. We need to add this tag to have a successive series of images. 

Image Orientation Patient

This tag is defined based on image coordinates and pixels. It defines the orientation of the image depending on the direction of the first line and the first column during the patient examination. The spectroscopic cartography images should have the same values as the patient orientation. We add this tag with the same defined values.

Number of Images in a DICOM Package

As a result of an MRI examination, DICOM images are classified into series. Each series contains images sharing the same sequence and same plane. Metabolic cartography on S-MRI is arranged in one file. To be accepted by LGP, this folder should contain at least three images.

Spacing Between Slices

This tag is the thickness of an axial slice. It represents the distance between two successive images. By modifying this tag, we concluded that the LGP-accepted distance between two DICOM images should be less than 1.5 mm. Spectroscopic cartography does not indicate this value. We should define a “space between slices” tag. The value must be equivalent to or less than 1.5 mm to be compatible with LGP. We adopted 1.5 mm as our default value. The acquisition of metabolic data on MRI accounts for this spacing value.

Photometric image: DICOM images obtained in spectroscopic cartography are coded in the “RGB (red, green, blue) scale” with 24 bits per pixel. LGP supports 16 bits per pixel, which create an incompatible situation. These 16 bits correspond to “monochrome 2” (i.e., grayscale). As an action, we reduce the bits/pixel on the spectroscopic cartography image by a post-treatment step. We applied a “16 bits monochrome 2” on 3D spectroscopic cartography.

Automation

**Figure 1:**
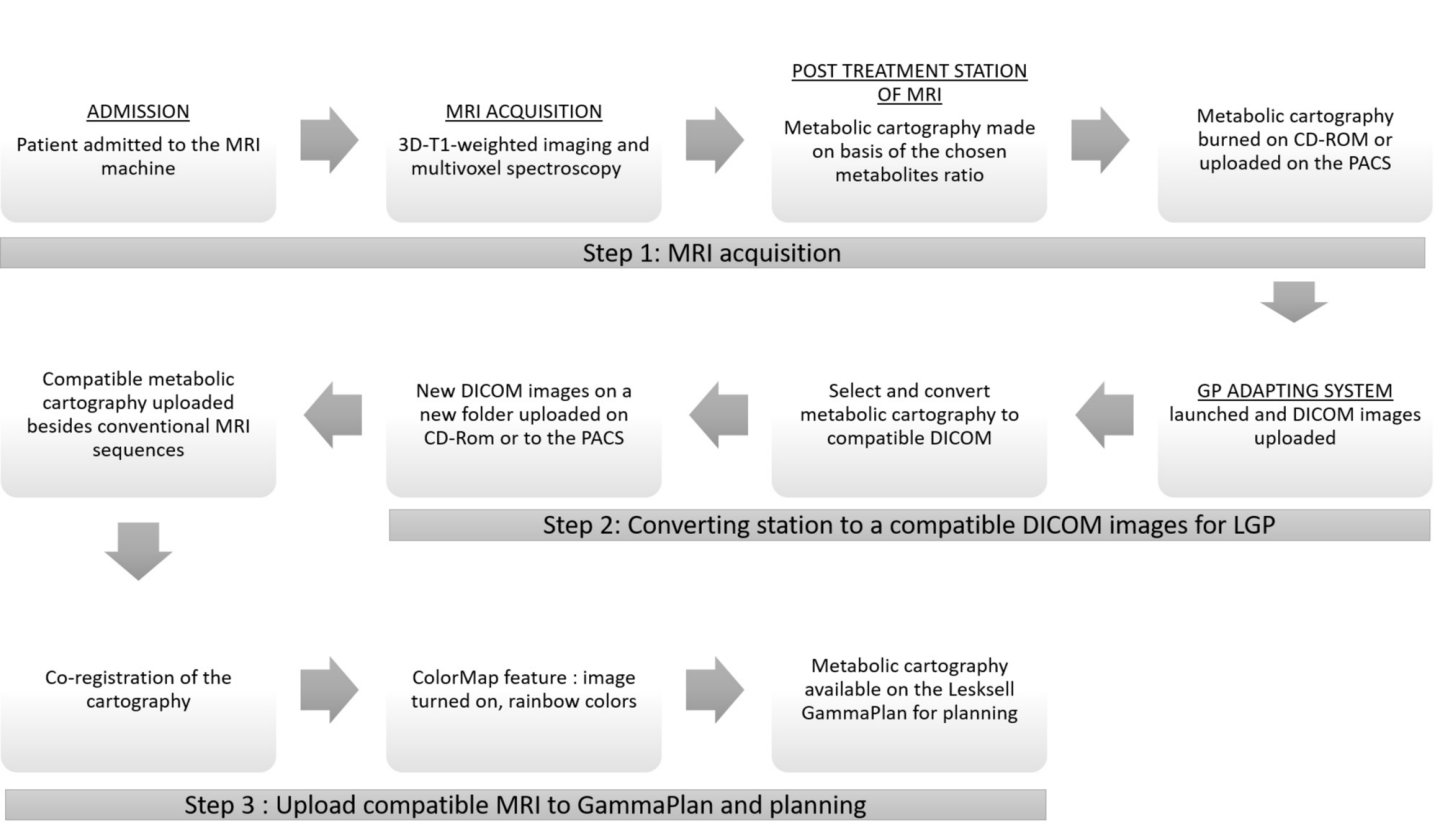
Flow chart of conversion and integration of an MRI metabolic cartography on with an LGP radiosurgical planning station MRI, magnetic resonance imaging; 3D, three-dimensional; CD-ROM, compact disc read-only memory; PACS, picture archiving and communications systems; DICOM, Digital Imaging and Communications in Medicine.

By performing these modifications, we could use a spectroscopic cartography image in radiosurgical planning by LGP. However, the process is time-consuming and non-reproducible by a radiosurgeon and could be a source of errors. We decided to create a software to reproduce these modifications behind a simple and rapid interface. We used Python 2.7.12 [14] to create a software that we call “GP Adapting Solution.” This software will execute all the protocols that we had established during the methodology steps (Figure 1).

## Results

GP Adapting Solution is compatible with Microsoft Windows XP and higher, and Linux Ubuntu 12.04 and higher. The processor should be at least Pentium 4 with a memory of at least 512 Mb.

**Figure 2:**
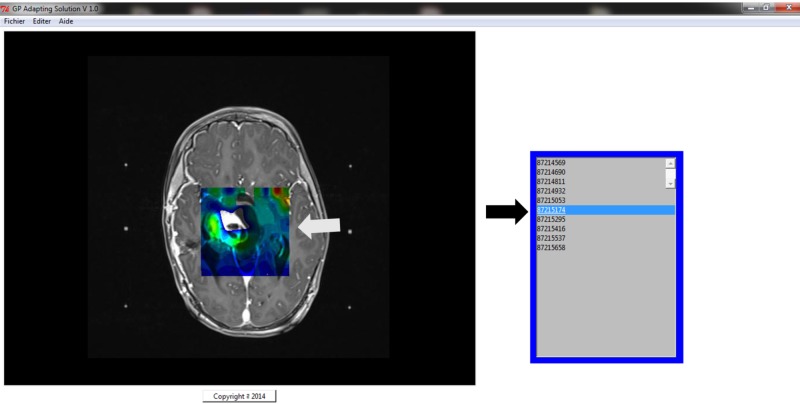
Snapshot of the GP Adapting Solution The menu in the upper part of the screen allows for the selection and conversion of the DICOM sequences. We can visualize all DICOM images by selecting the display part on each image. The white arrow indicates the visualization box, and the black arrow indicates the display list of DICOM images. GP, GammaPlan; DICOM, Digital Imaging and Communications in Medicine.

The GP Adapting Solution software allows the radio-surgeon to define the folder of MRI cartography and select files to be placed in one package. These images could be visualized and selected on this post-treatment station. In one click, the software will create a new folder named “patientname-date” and will include all the transformed images in this file. We can import the data on writable media or upload the data to PACS (picture archiving and communication system) [15] using an intranet (Figure 2).

**Figure 3:**
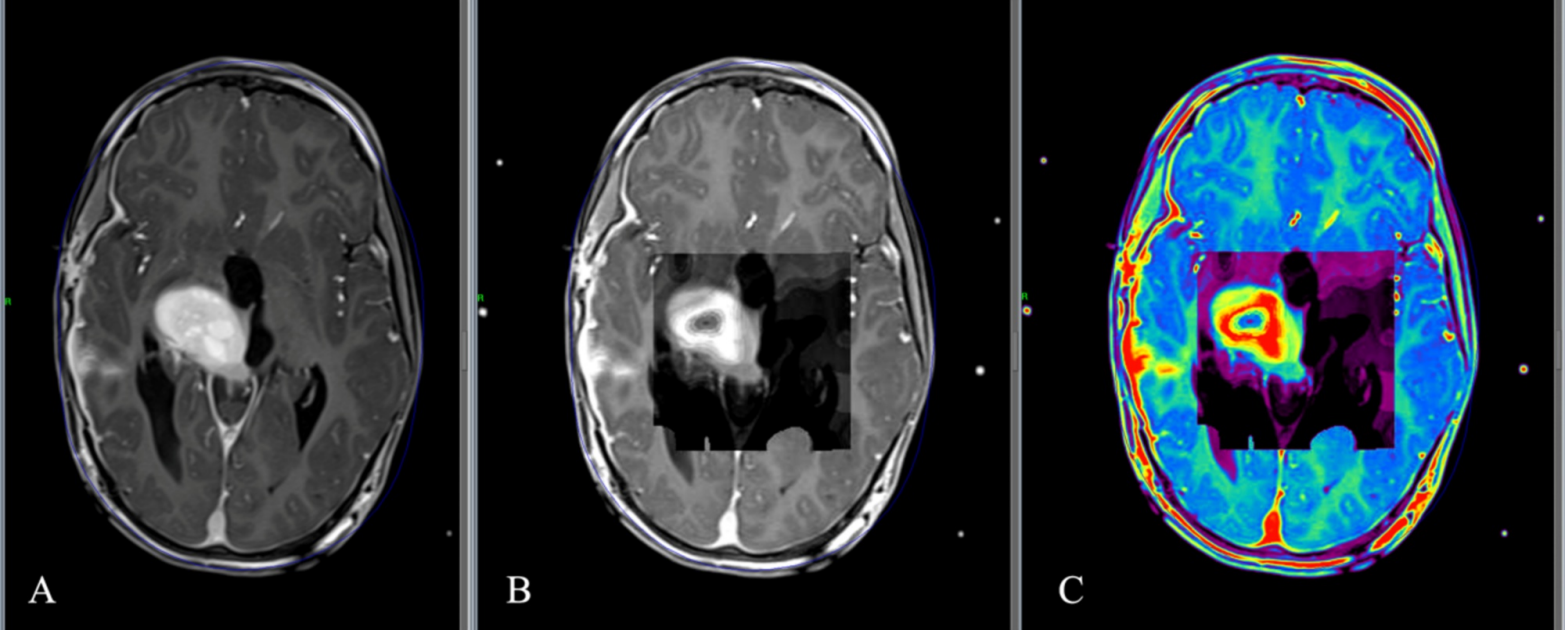
Integration of the metabolic cartography on LGP 10 Radiosurgical planning on 3D T1-weighted imaging (A). The metabolic cartography made based on multivoxel spectroscopic MRI is inserted on LGP using the GP Adapting Solution. This cartography is seen in grayscale (B). We select the image, click on the right button of the mouse, and then choose “colormap” on the menu. By selecting the “rainbow” feature, we obtain a colored image corresponding to the color code initially selected on the post-treatment station of MRI. Note that the red part of the tumor is where the choline/NAA ratio is the highest. 3D, three-dimensional; LGP, Leksell GammaPlan; GP, GammaPlan; MRI, magnetic resonance imaging; NAA, N-acetylaspartate.

Once the data are uploaded on the LGP inbox, we can use it in radiosurgical planning. As previously mentioned, the compatible cartography is in grayscale. We can restore the image color using a feature in the LGP by selecting the image, right-clicking on it, and then selecting “colormap” from the menu. We then change the option from grayscale to a rainbow. The image will appear in multiple colors like the original uploaded one. Finally, we can continue with radiosurgical planning according to the color-coding defined during the preparation of the metabolic cartography on the MRI post-treatment station depending the choline/N-acetylaspartate (NAA) ratio (Figure 3).

## Discussion

The primary goal of Gamma Knife radiosurgery is to deliver a high dose of irradiation in one session with high levels of accuracy. Only a high dose of irradiation can achieve the desired lesion control, especially in malignant tumors. This high dose cannot be offered by external beam radiotherapy due to the harmful effects on the surrounding healthy tissue.

Moreover, the limitation with radiosurgery is that the planning stage defines the enhancing lesion as a target, unlike the large target of radiotherapy. It is crucial to define the correct target. Conventional MRI could not delineate the real lesional volume nor the infiltration zone [16]. Unfortunately, this inhibits the application of Gamma Knife radiosurgery to some lesions such as gliomas and metastases given that the technique cannot accurately define these radiosurgical targets [17-18].

Proton magnetic spectroscopy

**Figure 4:**
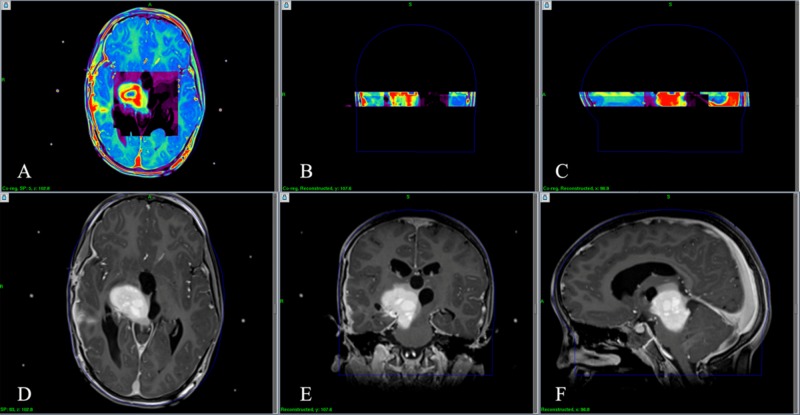
Uploading multivoxel spectroscopy on LGP 10 The volume studied by the multivoxel spectroscopy is uploaded in 3D planes on LGP. We can execute planning by 3D metabolic cartography (panel A represents the axial plane, panel B represents the coronal plane, and panel C represents the sagittal plane) and 3D T1-weighted imaging concurrently (panel D represents the axial plane, panel E represents the coronal plane, and panel F represents the sagittal plane). 3D, three-dimensional; LGP, Leksell GammaPlan.

The boundaries between glioma and normal tissue are vague, and the utility of conventional MRI is limited in this situation [18-20]. MRI spectroscopy is an imaging technique that considers the chemical change of a tumoral lesion. This change is visualized by spectral graphics as a result of a 3D analysis of multivoxel examination. By choosing one chemical or a ratio of two chemicals, we can have different representations of the metabolic behavior of a lesion [17,20]. As a result, we have metabolic cartography or 3D proton magnetic resonance spectroscopy (1H-MRS; Figure 4).

The MRI spectroscopy of glioma is characterized by a choline increase, NAA decrease, moderate creatine reduction, and lactate and lipid peaks for high-grade gliomas (HGG) [18,20]. McKnight et al. studied the choline/NAA index (CNI) for distinguishing the tumor from nontumorous tissue in patients suspected of having a glioma and found the sensitivity was 90% and specificity was 85% if CNI is >2.5 [21]. Another study reported that a CNI of >1.6 corresponds to sensitivity and specificity of 74.2% and 62.5%, respectively. The positive predictive value was 85.6% [20,22].

The usefulness of MRI spectroscopy for tumor targeting

In our daily practice, the radiosurgical target volume is delineated from contrast enhancement on a T1-weighted MRI where there is a compromised blood-brain barrier. The enhancement can be seen in an active tumor, where necrosis and radiation-induced changes are evident from previous therapies [21].

Chan et al. studied 26 patients treated by Gamma Knife radiosurgery after previous external beam radiotherapy of gliomas of World Health Organization grade IV [23]. The authors compared the radiosurgical target volume with the volume of the metabolically active tumor defined on 1H-MRS. This cohort was divided into two groups based on risk. The low-risk group patients had an overlap of less than 50% with respect to the metabolic lesion volume; the high-risk group had an overlap greater than 50%. The median survival was 15.7 months for the low-risk group and 10.4 months for the high-risk group [23]. The difference was statically significant (p < 0.01). A comparison of the radiosurgical irradiated target and metabolic lesion volume indicates a statistically significant difference. This result assumes the possible inclusion of necrosis, gliosis, and edematous tissue in addition to the tumor in the radiosurgical target volume. However, there is a possible underestimation of the target volume if we do not include the non-enhanced and metabolically abnormal parts of the lesion. Chan et al. concluded that the inclusion of 1H-MRS imaging might be beneficial in the treatment planning process [23]. 

This integration was also tried in different treatment modalities including surgery and radiotherapy. Zhang et al. conducted a study on the integration of 3D MRS maps on intraoperative navigation for glioma margin delineation, accounting for CNI thresholds for both low-grade gliomas (LGG) and HGG to achieve surgical resection [24]. The results confirmed the possibility of integration of 1H-MRS with conventional imaging in the perioperative surgical neuronavigation during surgical resection. A one-year follow-up indicates that glioma resection based on 3D MRS maps may contribute to a better prognosis (no recurrence for LGG and one recurrence for HGG).

Integration of 3D MRS in the radiosurgical treatment of tumors

Shen et al. used 1H-MRS to delineate gliomas for Gamma Knife radiosurgery [20]. The irradiation target was defined based on short T1, long T2, and CNI > 1.6. No additional software or post-processing station was reported to have been used to fuse conventional MRI and MRS data on the radiosurgical planning station. The authors observed that the addition of spectroscopic data decreases the maximum diameter of the target volume (p < 0.022). They concluded that MRS facilitates Gamma Knife radiosurgery using the actual and estimated lesion extent as the target volume [20].

In our report, we presented the possibility of integrating 3D MRS data on the planning station on the same screen with T1-weighted imaging with contrast enhancement by developing new software that we called the GP Adapting Solution. We offer a simple software solution that rewrites the metabolic cartography to meet LGP requirements. After that, the LGP will allow the fusion of the complete imported data. As a result, the delineation of a lesion is made based on a visualized metabolic abnormality integrated with tumoral activity on the radiosurgical planning station.

## Conclusions

We propose a new, simple, and open-source solution further facilitate the selection of a tumoral target for radiosurgical treatment with sufficient accuracy for radiosurgery. The development of this software solution may change the concept of radiosurgical management of brain lesions. The next step will be to apply this software in daily practice to assess the role of metabolic data in changing the prognosis of many malignant tumors. A prospective multicenter study is warranted to confirm this hypothesis.

## References

[1] Levivier M, Wikler D, Massager N (2002). The integration of metabolic imaging in stereotactic procedures including radiosurgery: a review. J Neurosurg.

[2] MAGNETOM Avanto eco. (2019) (2019). MAGNETOM Avanto eco. https://www.siemens-healthineers.com/en-us/magnetic-resonance-imaging/0-35-to-1-5t-mri-scanner/magnetom-avanto.

[3] 3] Leksell Gamma Knife® Perfexion™. (2019 (2019). Leksell Gamma Knife® Perfexion™. https://www.elekta.com/radiosurgery/leksell-gamma-knife-perfexion/#lgk-icon-ar.

[4] (2019). Leksell GammaPlan®10. https://www.elekta.com/radiosurgery/leksell-gammaplan/leksell-gammaplan-10.html.

[5] Bell W (2007). Reverse Engineering. Global Media Publications, Delhi.

[6] Yushkevich PA, Gao Y, Gerig G (2016). ITK-SNAP: an interactive tool for semi-automatic segmentation of multi-modality biomedical images. Conf Proc IEEE Eng Med Biol Soc.

[7] 7] Softpedia (2019). Softpedia. DCM Compare 3.0.3. http://www.softpedia.com/get/Science-CAD/DCM-Compare.shtml.

[8] Escott EJ, Rubinstein D (2003). Free DICOM image viewing and processing software for your desktop computer: what’s available and what it can do for you. Radiographics.

[9] Escott EJ, Rubinstein D (2004). Informatics in radiology (infoRAD): free DICOM image viewing and processing software for the Macintosh computer: what’s available and what it can do for you. Radiographics.

[10] National Electrical Manufacturers Association (2015). Determination of image uniformity in diagnostic magnetic resonance images. NEMA Standards Pub.

[11] National Electrical Manufacturers Association (2018). Determination of slice thickness in diagnostic magnetic resonance imaging. NEMA Standards Pub.

[12] National Electrical Manufacturers Association (2015). Determination of two-dimensional geometric distortion in diagnostic magnetic resonance images. NEMA Standards Pub.

[13] National Electrical Manufacturers Association (2017). Quantification and mapping of geometric distortion for special applications. NEMA Standards Pub.

[14] 14] Python Software Foundation. (2019 (2019). Python Software Foundation. https://www.python.org.

[15] Strickland NH (2000). PACS (picture archiving and communication systems): filmless radiology. Arch Dis Child.

[16] Nelson SJ (2011). Assessment of therapeutic response and treatment planning for brain tumors using metabolic and physiological MRI. NMR Biomed.

[17] Bieza A, Krumina G (2013). The value of magnetic resonance spectroscopy and diffusion tensor imaging in characterization of gliomas growth patterns and treatment efficiency. J Biomed Sci Eng.

[18] Horská A, Barker PB (2010). Imaging of brain tumors: MR spectroscopy and metabolic imaging. Neuroimaging Clin N Am.

[19] Burger PC, Heinz ER, Shibata T, Kleihues P (1988). Topographic anatomy and CT correlations in the untreated glioblastoma multiforme. J Neurosurg.

[20] Shen G, Xu L, Xu M, Geng M, Tan Y, Li F (2012). 1H-MR spectroscopy guided gamma knife radiosurgery for treatment of glioma. Turk Neurosurg.

[21] McKnight TR, von dem Bussche MH, Vigneron DB (2002). Histopathological validation of a three-dimensional magnetic resonance spectroscopy index as a predictor of tumor presence. J Neurosurg.

[22] Pirzkall A, McKnight TR, Graves EE (2001). MR-spectroscopy guided target delineation for high-grade gliomas. Int J Radiat Oncol Biol Phys.

[23] Chan AA, Lau A, Pirzkall A (2009). Proton magnetic resonance spectroscopy imaging in the evaluation of patients undergoing gamma knife surgery for Grade IV glioma. Spec Suppl.

[24] Zhang J, Zhuang D-X, Yao C-J (2016). Metabolic approach for tumor delineation in glioma surgery: 3D MR spectroscopy image-guided resection. J Neurosurg.

